# High paediatric HIV viral load rejection rate in South Africa, a retrospective review

**DOI:** 10.4102/sajhivmed.v26i1.1687

**Published:** 2025-03-31

**Authors:** Tanya Y. Murray, Gayle G. Sherman, Dumisani Mlotshwa, Ahmad F. Haeri Mazanderani

**Affiliations:** 1 Centre for HIV and STIs, National Institute for Communicable Diseases, National Health Laboratory Service, Johannesburg, South Africa; 2 Department of Paediatrics and Child Health, Faculty of Health Sciences, University of the Witwatersrand, Johannesburg, South Africa

## Introduction

In South Africa, there are an estimated 160 000 children under the age of 15 years living with HIV, of whom 63% were on antiretroviral treatment (ART) in 2023.^1^ HIV viral load (VL) testing is the preferred approach for monitoring response to treatment,^2^ but there are concerns regarding equitable access to VL testing for children,^3^ with challenges including capacity for paediatric specimen collection.^4^ HIV VL monitoring is vital for improving patient outcomes and achieving Joint United Nations Programme on HIV/AIDS (UNAIDS) 95-95-95 targets.^5^

The National Health Laboratory Service (NHLS) is the public sector provider of clinical laboratory services in South Africa, serving 80% of the population. Their laboratory services include HIV VL testing, which is conducted centrally at 17 laboratories, using the Roche Cobas^®^ HIV-1 Quantitative nucleic acid test on the Cobas^®^ 6800/8800 Systems (Roche Diagnostics GmbH, Mannheim, Germany) and Abbott Alinity m HIV-1 assay (Abbott Molecular Inc, Des Plaines, Illinois, United States) during 2022 and 2023.^6^ HIV VL testing is done only on plasma specimens, with a recommended whole blood volume of 5 mL in a plasma preparation tube (PPT).^7^ For infants, 500 µL ethylenediaminetetraacetic acid (EDTA) whole blood (in a 0.5 mL EDTA microtainer collection tube) is acceptable, requiring the assay be run in dilution.

The NHLS has essential criteria for specimen acceptance for laboratory testing to ensure the integrity of test results. Specimens may be rejected if these criteria are not met, and examples of rejection criteria include inadequate specimen volume, incorrect specimen container, and incomplete request form submitted with a specimen.^7,8^ For all tests conducted within the NHLS, the acceptable rejection rate threshold is < 3%.^9^ While several studies have analysed specimen rejection rates and reasons at the NHLS,^8,10,11^ none has focused on HIV VL testing in children. The aim of this analysis was to determine the rejection rates for specimens submitted to the NHLS for HIV VL testing among children in South Africa. Rejection reasons were also described.

## Methods

A retrospective descriptive analysis of routine laboratory data from South Africa’s public health sector was conducted. Data were extracted from the National Institute for Communicable Diseases (NICD) Data Warehouse in September 2023, for HIV VL specimens registered and rejected within the NHLS from 01 June 2022 to 31 May 2023. Extracted variables included geographical location (province), age (categorised into ≥ 15 years; < 15 years and < 5 years [a subset of < 15 years]), specimen type, and rejection reasons (predefined list on the laboratory information system [LIS]). Rejection rates were calculated using total HIV VL samples registered as the denominator and described for all ages, < 15-year-olds and < 5-year-olds. Rejection reasons, as per the LIS, were further grouped into nine broader categories and described for < 15-year and < 5-year age groups.

## Results

The overall rejection rate for the 1-year period in all ages was 3% (203 694 HIV VL rejections/6 741 118 HIV VL tests registered). In the < 15-year-olds, the rejection rate was 6% (9850 rejections/173 768 VL tests), and in the < 5-year-olds, it was 13% (4489 rejections/35 818 VL tests).

Over the 12-month period, there were 122 rejection reasons provided on the LIS for all ages, of which 74 included reasons for children < 15 years. Overall, the main rejection reason was ‘haemolysed specimen’, accounting for 25% of all rejections (51 731/203 694). Of the 74 reasons provided for < 15-year-olds, six rejection reasons accounted for 83% of all rejections both among < 15-year-olds (8158/9850 rejections) and among < 5-year-olds (3730/4489 rejections). The main reason for paediatric VL rejection was ‘insufficient specimen’ (26%, *n* = 2556 in < 15 years; 35%, *n* = 1577 in < 5 years), followed by ‘haemolysed specimen’ (25.5%, *n* = 2514 in < 15 years; 18%, *n* = 807 in < 5 years). The remaining four of the six main reasons were ‘require PPT specimen’ (12%, *n* = 1190 in < 15 years; 13%, *n* = 578 in < 5 years), ‘cancel by gatekeeping’ (7%, *n* = 723 in < 15 years; 4.5%, *n* = 201 in < 5 years), ‘specimen insufficient for rerun’ (6%, *n* = 615 in < 15 years; 6.5%, *n* = 294 in < 5 years), and ‘specimen not received’ (6%, *n* = 560 in < 15 years; 6%, *n* = 273 in < 5 years). Additional analysis of the ‘require PPT specimen’ rejection reason indicated that specimen types captured on the LIS with this rejection code were nearly all registered as PPT specimens (99%, *n* = 1182 in < 15 years; 99%, *n* = 575 in < 5 years), with minimal unknown (1%, *n* = 7 in < 15 years; 1%, *n* = 3 in < 5 years) and EDTA (*n* = 1 in < 15 years).

**FIGURE 1:**
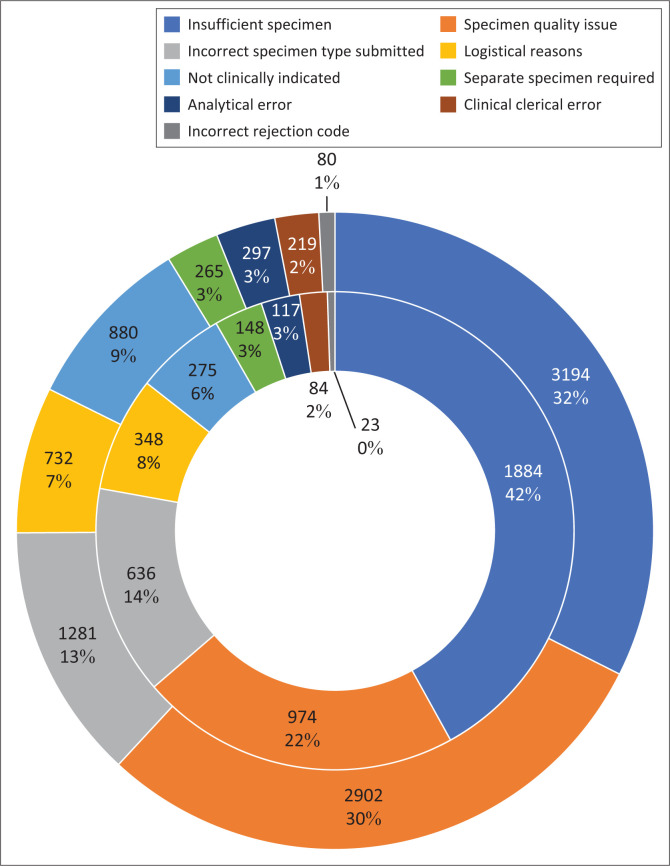
National HIV viral load rejection categories in children < 5 years (*n* = 4489; inner doughnut) and < 15 years (*n* = 9850; outer doughnut), 01 June 2022 to 30 May 2023.

For further analysis by age group and geographical location, the 74 rejection reasons were grouped into nine broad rejection categories, listed as follows (with the six main rejection reasons indicated in brackets): (1) analytical error, (2) clinical clerical error, (3) incorrect rejection code, (4) incorrect specimen type submitted (e.g. ‘require PPT specimen’), (5) insufficient specimen (e.g. ‘specimen insufficient’, ‘specimen insufficient for rerun’), (6) logistical reasons (e.g. ‘specimen not received’), (7) not clinically indicated (e.g. ‘cancel by gatekeeping’), (8) separate specimen required, and (9) specimen quality issue (e.g. ‘unsuitable: haemolysed’). The three rejection categories that accounted for > 75% of rejections in < 15-year-olds and < 5-year-olds, were related to the specimen collection process (Figure 1).

**FIGURE 2:**
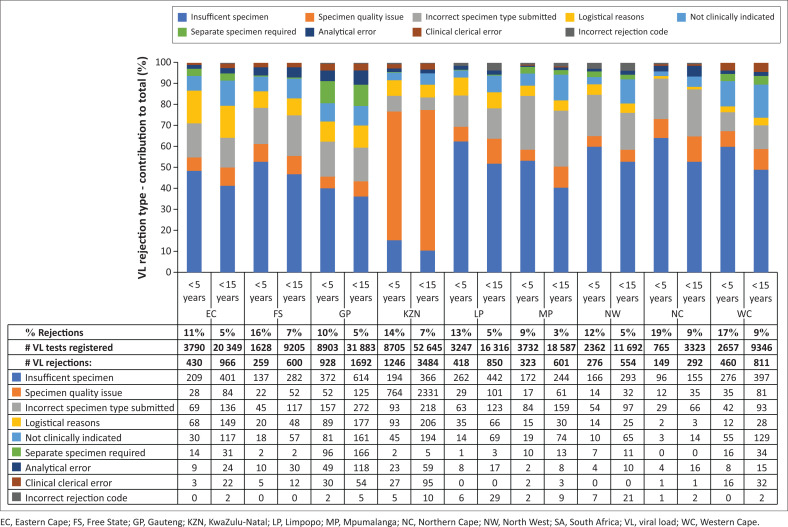
HIV viral load rejection rates and reasons per province in children < 5 years and < 15 years, 01 June 2022 to 31 May 2023.

Rejection rates varied per province (Figure 2). Among specimens from < 15-year-olds, rejection rates ranged from 3% (*n* = 601) in Mpumalanga (MP) to 9% (*n* = 292) in the Northern Cape (NC) and 9% (*n* = 811) in the Western Cape (WC). For the < 5-year-olds, rejection rates were higher, ranging from 9% (*n* = 323) in MP to 19% (*n* = 149) in NC. Reasons for the majority of rejected specimens were similar across the provinces, with insufficient specimen being the most frequent rejection reason in all but one province. The exception was KwaZulu-Natal (KZN), where specimen quality issues (haemolysed specimens) were most common, accounting for 80% (2331/2902) of all specimen quality rejections among < 15-year-olds in the country.

## Discussion

The overall (all ages) HIV VL rejection rate for specimens submitted to the NHLS over the 1-year period was acceptable, at 3%. However, there was a notably higher HIV VL rejection rate among paediatric patients (< 15-year-olds), at 6%, which was higher still among < 5-year-olds, at 13%.

Nationally, the main reason for paediatric HIV VL specimen rejection was insufficient specimen volume (32%), and this proportion was even higher when restricted to specimens from < 5-year-olds (42%). The next two main reasons were related to specimen quality issues (30% among < 15-year-olds; 22% among < 5-year-olds), and incorrect specimen type submitted (13% among < 15-year-olds; 14% among < 5-year-olds). This suggests an overall challenge with the clinical process of paediatric HIV VL specimen collection. However, there was some variability in the reasons for specimen rejection across provinces, suggesting a more targeted approach to improving VL rejection rates may be required to optimally improve performance. For example, specimens rejected because of haemolysis occurred almost exclusively in KZN, which requires further investigation. Another concern was the high number of rejections on account of an incorrect specimen type submitted, with the predominant rejection reason on the LIS being ‘Require PPT specimen’. As almost all such rejections were registered as PPT specimens, there is a clear need to improve the quality of data capturing relating to specimen type within the NHLS.

Paediatric phlebotomy is a global challenge,^12^ and generally results in higher pre-analytical rejection rates than in adults.^13^ The results of this analysis suggest that the majority of paediatric HIV VL rejections relate to pre-analytical errors (i.e., prior to the specimen being processed in the laboratory) – a finding which is consistent with prior reports evaluating NHLS rejection data for CD4 testing, HIV serology, and general blood sampling.^8,10,11^ Ensuring sufficient specimen volume and correct sampling procedures would address the majority of such rejections. Where blood specimen collection by venipuncture is inaccessible, capillary blood draws by finger- or heel-prick may be an option for collecting 0.5 mL.^14^ Plasma specimens remain the only sample type processed for HIV VL testing within the NHLS. Although dried whole blood spot (DBS) specimens are approved by the WHO as an alternative specimen type in cases where plasma-based VL testing is hindered by logistical, infrastructural or operational obstacles,^3^ DBS specimens are currently not accepted for HIV VL testing by the NHLS on account of inaccurate HIV RNA quantitation below 1000 copies/mL.^15^

Importantly, the high volume of paediatric HIV VL test rejections represents the tip of the iceberg regarding gaps within the overall paediatric HIV care cascade. Only 63% of children living with HIV are estimated to be on ART,^1^ of which approximately only three-quarters have HIV VL monitoring (Haeri Mazanderani A, 2024, personal communication, November 21). Children receiving ART but not having HIV VL monitoring may relate to the lack of paediatric blood sampling skills at clinic level. This is supported by our finding of a high proportion of pre-analytical rejections. HIV VL testing is the recommended approach to monitor treatment response among infants and children,^4^ and in order to achieve this, it is essential that specimens from children are of acceptable quality for testing. The particularly high HIV VL specimen rejection rate in children negatively impacts their quality of care. Strengthening phlebotomy skills presents the most effective strategy for maximising a reduction in VL rejections, and evaluating routine laboratory data can support monitoring the effectiveness of quality improvement projects relating to this.
